# Burden of Serious Fungal Infections in Jordan

**DOI:** 10.3390/jof4010015

**Published:** 2018-01-18

**Authors:** Jamal Wadi, David W. Denning

**Affiliations:** 1 The Medical Center, Jordan Hospital and Medical Center, 29 Adeeb Wahbeh Street, 11118 Amman, Jordan; 2 Division of Infection, Immunity and Respiratory Medicine, Faculty of Biology, Medicine and Health, The University of Manchester, Manchester M23 9LT, UK; david.denning@manchester.ac.uk

**Keywords:** mycoses, epidemiology, aspergillosis, candidemia, fungal infections

## Abstract

Objective: To estimate the burden of fungal infections in Jordan for the first time. Material and Methods: Population data was from UN 2011 statistics and TB cases from WHO in 2012. Fewer than 100 patients with HIV were recorded in Jordan in 2013. Approximately 100 renal transplants and eight liver transplants are performed annually. There were 12,233 major surgical procedures in Jordan in 2013, of which 5.3% were major abdominal surgeries; candidemia was estimated in 5% of the population based on other countries, with 33% occurring in the ICU. *Candida* peritonitis/intra-abdominal candidiasis was estimated to affect 50% of the number of ICU candidemia cases. No adult asthma rates have been recorded for Jordan, so the rate from the Holy Land (8.54% clinical asthma) from To et al. has been used. There are an estimated 49,607 chronic obstructive pulmonary disease (COPD) patients in Jordan, with 64% symptomatic, 25% Gold stage 3% or 4%, and 7% (3472) are assumed to be admitted to hospital each year. No cystic fibrosis cases have been recorded. Literature searches on fungal infections revealed few data and no prevalence data on fungal keratitis or tinea capitis, even though tinea capitis comprised 34% of patients with dermatophytoses in Jordan. Results: Jordan has 6.3 million inhabitants (65% adults, 6% are >60 years old). The current burden of serious fungal infections in Jordan was estimated to affect ~119,000 patients (1.9%), not including any cutaneous fungal infections. Candidemia was estimated at 316 cases and invasive aspergillosis in leukemia, transplant, and COPD patients at 84 cases. Chronic pulmonary aspergillosis prevalence was estimated to affect 36 post-TB patients, and 175 in total. Allergic bronchopulmonary aspergillosis (ABPA) and severe asthma with fungal sensitization (SAFS) prevalence in adults with asthma were estimated at 8900 and 11,748 patients. Recurrent vulvovaginal candidiasis was estimated to affect 97,804 patients, using a 6% rate among women 15–50 years of age. Conclusion: Based on local data and literature estimates of the frequency of mycoses in susceptible populations, at least 1.9% of Jordanians have some form of serious fungal disease.

## 1. Introduction

**Figure 1:**
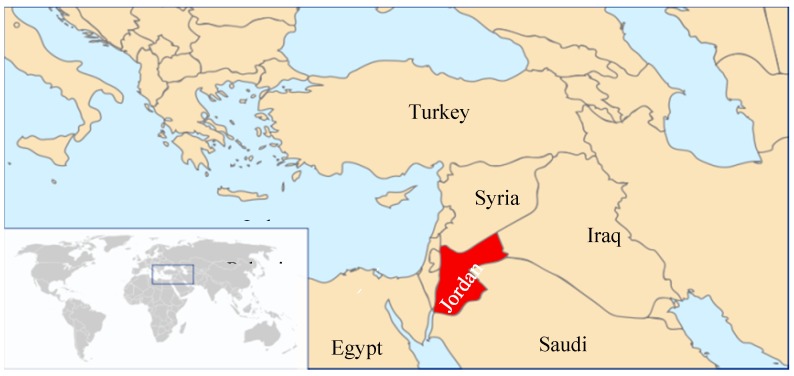
Map of Jordan, location in relation to nearby countries, and the world as in the inset (modified from [5]).

Jordan and the surrounding region have scanty information on the population prevalence of fungal infections. Jordan is a small country with 6.3 million inhabitants (65% adults, 6% are >60 years old) [1,2]. It is in the Eastern Mediterranean region (Figure 1), east of Palestine and west of Iraq and Saudi Arabia, in a relatively moderate climate where populations are mostly concentrated in the mountains, and less populated in the eastern part of the country where desert predominates [3]. This epidemiological modeling study is designed to assess the burden of serious fungal infections in Jordan based on some local data and data obtained from the UN 2011 and WHO 2013 for Jordan. The burden of serious fungal infections, their prevalence, as well as their trends, have not been studied before in Jordan, and the information on this issue continues to be speculative. A study from Jordan by Abu-Elteen and Abdul Malek assessing cutaneous mycosis and their causative species was published in 1999 [4].

Study of the burden of serious fungal infections using epidemiological surveillance data is rarely possible worldwide as data is scarce but, if done, it is mostly based on laboratory culture and autopsy data [6]. Studying the fungal burden and species distribution in a community is of a principal importance as these infections cause high morbidity and mortality, especially in special risk groups, such as critically ill ICU patients subjected to several interventions, including many broad-spectrum antibiotics, central venous catheters, total parenteral nutrition, hemodialysis, and ventilators; patients with abdominal surgery; cancer patients with neutropenia, hematological diseases, and/or transplants; patients receiving solid organ transplants, patients with different immunosuppressive states, or on immunosuppressive therapy, and patients with chronic respiratory diseases [7,8]. Summarizing all the data in any region, including this region, empowers proper nationwide planning to minimize death and suffering from fungal disease, and can guide attending healthcare professionals on appropriate empirical approaches in complex patients with possible, probable, or confirmed serious fungal infections [9].

Our current study depicts the current state of epidemiological knowledge and estimates the incidence and prevalence of serious fungal infections in Jordan.

## 2. Materials and Methods

We collected data on total and at risk populations. Population data was from UN 2011 statistics and from the WHO in 2012, supported by crudely locally-collected data. Fewer than 100 patients with HIV were recorded in Jordan in 2013. Approximately 100 renal transplants and eight liver transplants are performed annually. There were 12,233 major surgical procedures in Jordan in 2013, of which 5.3% were major abdominal surgeries; candidemia was estimated in 5% of the population based on other countries, with 33% occurring in ICU. *Candida* peritonitis/intra-abdominal candidiasis was estimated to affect 50% of the number of ICU candidemia cases. No adult asthma rates have been recorded for Jordan, so the rate in adults from the Holy Land (8.54% clinical asthma) from To et al. has been used [10]. There are an estimated 49,607 chronic obstructive pulmonary disease (COPD) patients in Jordan [11], with 64% symptomatic, 25% Gold stage 3 or 4 [12], and 7% (3472) are assumed to be admitted to hospital each year. Tuberculosis rates have risen slightly over the last few years and we used the WHO 2015 estimates for pulmonary TB only assuming a 10% mortality [13]. Pulmonary tuberculosis was assumed to represent 20% of the total of chronic pulmonary aspergillosis cases [14]. No cystic fibrosis cases have been recorded. Literature searches on fungal infections revealed little data and no prevalence data on fungal keratitis or tinea capitis, even though tinea capitis comprised 34% of patients with dermatophytoses in Jordan [4].

### Data Extraction and Statistics

Data were collected from several hospitals, Jordan Nephrology Society. Cancer book statistics, and “Al Mashoura”, the sole HIV clinic in Jordan, with assumptions based on literature review, and simulating from other countries data for special risk groups. Cancer rates were extracted based on published data by WHO Regional Office for the Eastern Mediterranean (WHO-EMRO) and Jordan ministry of health, and both data were similar for cancer prevalence [15,16]. Models of the data were based on a literature review and simulation. Rates were calculated per 100,000 population, and compared with data available from other regional countries, like Iraq, Egypt, Algeria, and Saudi Arabia, and the estimated available data from many other countries.

## 3. Results

Jordan has 6.3 million inhabitants, about 4.2 million (65%) adults, mostly in the lower age categories, and 6% are >60 years old. The current burden of serious fungal infections in Jordan was estimated to affect ~119,000 patients (1.9%), not including any cutaneous fungal infections (Table 1). Candidemia burden was estimated at 316 cases, and invasive aspergillosis at 84 cases in patients with cancer and ICU [17], transplant, and COPD patients at 84 cases. In HIV patients only, the esophageal candidiasis rate was 0.10/100,000 [14,17], and oral candidiasis 0.14 [18], adding cancer patients as a risk group made estimates for oral candidiasis about 0.22/100,000 and esophageal candidiasis about 0.21/100,000 ([17,19,20,21,22,23], and EMRO 2012 [15], many more cases occur in other risk groups, but we are unable to estimate these numbers with confidence. Chronic pulmonary aspergillosis (CPA) prevalence was estimated to affect 36 post-TB patients, and 175 in total [24]. Allergic bronchopulmonary aspergillosis (ABPA) and severe asthma with fungal sensitization (SAFS) prevalence in adults with asthma were estimated at 8900 and 11,748 patients [25]. Recurrent vulvovaginal candidiasis was estimated to affect 97,804 patients, using a 6% rate among women 15–50 years of age [26]. Endemic fungal infections, such as histoplasmosis, coccidioidomycosis, paracoccidioidomycosis, and blastomycosis, have never been reported in Jordan until the writing of this manuscript.

## 4. Discussion

The burden of serious fungal infections is expected to increase in Jordan, as more hospitals and ICUs are being established, combined with more invasive procedures and a growing immunocompromised population [27]. Jordan has a rapidly growing international healthcare market for advanced therapy of cancer and complex surgery. In addition, the civil war in Syria has brought large numbers of refugees into the country, some of whom have significant illnesses, such as tuberculosis. Our current study evaluated the burden of serious fungal infections collected from several sources and assumptions were made based on the available rates for the similar age and risk groups from different studies. Hence, these are empirical assumptions that can be used in the absence of better local data construed based on countrywide reporting/surveillance systems, when available.

### 4.1. Candidiasis

Comparing the Jordan rate (Table 1) with other regional countries (Table 2), like Saudi Arabia, candidemia rates were estimated to be higher in Saudi Arabia at 10/100,000, while the estimate for Jordan is 5/100,000 [28]; Algeria, Iraq, and Egypt candidemia rates were similar to Jordan and arbitrary, as no good epidemiological data are published (5/100,000) [29,30,31]. *Candida* peritonitis in Jordan was estimated to be 0.74/100,000, almost similar to Algeria and Egypt where both recorded rates of 0.75/100,000. Again, Saudi Arabia’s rate was double (1.6/100,000) that of Jordan. The *Candida* vaginitis rate in Jordan was 3097/100,000 and in Iraq, Saudi Arabia, Egypt, and Algeria were 2664, 3320, 3169, and 2402 per 100,000, respectively. Oral candidiasis in Jordan was estimated at 0.22/100,000 in the general population, and esophageal candidiasis 0.21/100,000 population [21,22,32]. Other Arab countries’ (Asia and Africa) rates are as follows: Iraq (0.26, 0.14), Egypt (2.73, 0.85), Algeria (-, 2.1), and Saudi Arabia (-, 1.4) per 100, 000 population, respectively, though the estimated rates for Egypt oral candidiasis is higher than others.

### 4.2. Aspergillosis

Several clinical presentations were estimated, including invasive aspergillosis (IA), allergic bronchopulmonary (ABPA), severe asthma with fungal sensitization (SAFS), and chronic pulmonary aspergillosis (CPA) [17,24,25]. Jordan’s rate for invasive aspergillosis was 1.34/100,000, the lowest estimate among other comparator countries of the region. In Iraq, the estimated rate of invasive aspergillosis was 2.26/100,000; in Saudi Arabia, 7.6/100,000; in Algeria, 7.1/100,000; and in Egypt, 10.7/100,000 (Table 2). The Jordan prevalence of CPA was estimated at 11/100,000, higher than Saudi Arabia (3.4/100.000) and Algeria (2.2/100,000), and similar to Iraq where the prevalence was estimated as low as 1.15/100,000. In Egypt the prevalence estimate was 13.83/100,000, the highest of all, while estimates reported from Iraq may have been an under-estimation. Asthma is relatively common in Jordan and, therefore, the prevalence of ABPA and SAFS were 141 and 186 per 100,000 population, respectively, close to rates in Egypt (162.15 and 214), but higher than Iraq (16 and 13) and Algeria (77 and 102), and lower than Saudi Arabia (212 and 280) per 100,000 population. We have been unable to estimate a burden estimate for allergic fungal rhinosinusitis.

### 4.3. Other Fungal Infections

Mucormycosis annual rates in Iraq and Algeria were estimated, as in Jordan, at about 0.2/100,000 [29,30,31], while 0.034/100,000 for Saudi Arabia (Table 2), whereas in Canada and France the rate was recorded at 0.12/100,000 [34,35], an earlier French study based on “capture recapture” methodology added (0.028/100,000), i.e., 23% more cases to the generally-known rates [36]. We do not believe any cases of cryptococcal meningitis have been recorded in Jordan; at least, from Jordan Hospital and Medical Center, Amman, Jordan one non-HIV related cryptococcal meningitis was diagnosed under JW’s (one of the authors) care about a year after this study was posted as an abstract in Copenhagen, 25th European Congress of Clinical Microbiology and Infectious Diseases (ECCMID 2015), EV0938 (personal knowledge). There are so few patients with HIV infection that *Pneumocystis jirovecii* pneumonia is probably uncommon or rare, and is probably more common in other immunocompromised patients. Neither disease was recorded in Saudi Arabia, respectively (Table 1 and Table 2) [28,29,31].

### 4.4. The Global Burden of Fungal Diseases

Other parts of the world showed diverse estimates for the rates of fungal infections, where about 68 countries were evaluated for the burden of fungal infections; they constitute about 80% of the world’s population [37,38]. The burden of fungal infections in India seemed to have had escalated in one of the largest populated countries of Asia and the world; invasive candidiasis was estimated 1–12 per 1000 hospital admissions. Most importantly, in a tertiary care center, invasive candidiasis had escalated several fold for the period 1980–1995 to rank fourth among blood isolates [39], like that observed in the United States for almost the same period [40]. China’s estimation of fungal infections contributes to the world with a heavy burden as their population creeps toward 1.4 billion [41], and China invasive candidiasis is estimated, like other parts of the world, including Jordan (5/100,000) and Canada (4.9/100,000) [34], to be lower than the rates estimated for the UK (8.14/100,00) and Spain (10.7/100,000) [42,43]. Esophageal candidiasis estimated rates in most of the world’s countries are higher than Jordan (0.21/100,000); Tanzania and Nigeria estimates were 203 and 93.2 per 100,000 population, respectively, probably due to high HIV patient burden in both countries; intermediate rates, like Vietnam, Guatemala, and Senegal (36, 29, and 14/100,000), respectively; while UK rates ranged from 0.07–0.58/100,000. The invasive aspergillosis estimate in China was 11.9/100,000, closer to Vietnam (15.99/100,000) [44], higher than Mexico (4.47/100,000) [45] and Guatemala (4.4/100,000) [46], and higher than the rates estimated for the UK (4.59-4.61/100,000), Spain (2.86/100,000), and Canada (1.9/100,000), as well as some African countries, like Nigeria (0.6/100,000) [47] and Tanzania (0.05/100,000) [48]. China’s rates for ABPA and SAFS are estimated at 36.1 and 47.6 per 100,000, respectively. Vietnam’s rates, at 26 and 34 per 100,000, both Asian countries were different from Nigeria and Senegal estimates of 60.5 and 79.9 and 71 and 93 per 100,000 population, respectively, in Africa [47,49], however, Australia’s rates were the highest estimates for ABPA and SAFS, at 245 and 323 per 100,000 population [50], closer to what was estimated in the UK with a range of 175–372 and 192–654 per 100,000 population [42], and Canada, with 172 and 277 per 100,000 population. Except for the estimated low mucormycosis rates in the UK (0.09/100,000) and Canada (0.19/100,000), other estimated world countries have similar rates (0.2/100,000 population). HIV/AIDS-related invasive fungal infection estimates are high in Nigeria and Tanzania, where *Cryptococcus* and *Pneumocystis* infections rated 37.4 and 48.2 and 14.66 and 22 per 100,000 population [47,48], whilst other estimated countries recorded rates ranging from 0.18 to 0.19 in Canada, 0.15 to 0.67 in Vietnam, and 2.6 to 8.2 in Senegal for *Cryptococcus* and *Pneumocystis* infections, respectively (Table 3) [34,44,49].

Invasive fungal infections carry considerable morbidity and mortality, and opportunistic fungal infections, including invasive aspergillosis and candidiasis, cryptococcosis, mucormycosis, and *Pneumocystis* pneumonia are associated with high mortality, but less with treatment, partly because of late diagnoses and treatment. Hitherto, many healthcare systems lack proper data on invasive fungal infections IFI, they are not incorporated in hospitals’ quality indicators, and there are no policies or guidelines drafted for their early clinical diagnosis and the appropriate use of the antifungal therapy, like what has been drafted (in few hospitals) for the diagnosis and treatment of some hospital-associated infections in Jordan. In addition to educating the concerned healthcare professionals on early clues on the diagnosis to consider early justified empirical treatment, especially in resource-limited countries where the cost of antifungal agents is prohibitive, there is hesitancy to start empirical antifungal therapy [36,51]. On the other hand, international organizations do not stress enough the importance of IFI, e.g., tuberculosis and malaria, though fungal disease morality is about as high as that from tuberculosis, 1.5 to 2.0 million annually, and WHO has only mycetoma as a focus for fungal infections as part of global efforts to reduce the impact of disease [52,53].

Chronic pulmonary aspergillosis, ABPA and SAFS are somehow amenable to antifungal treatment and affect tens of millions. The mortality estimate is about 400,000 from chronic pulmonary aspergillosis and a substantial proportion of the 350,000–480,000 who die of asthma each year. In Jordan, endemic serious fungal infections are rare [25] but, globally, add to the worldwide burden of more than 65,000 life-threatening infections; some have an associated mortality as high as 70% [54]. Jordan is also remarkable in having very few HIV-infected patients, but across the world nearly 50% of deaths from AIDS can be attributed to fungal infections. Local authorities in Jordan, i.e., the Ministry of Health, influenced by WHO, rightfully stress tuberculosis diagnosis, treatment, and community follow up, while not paying enough attention to IFI as a cause of morbidity and mortality. Authors and policy-makers frequently criticize the use of antibacterials in agriculture as a major contributor to bacteria resistance overlooking the environmental use of the azole antifungals in some countries, which contribute to the burden of resistant molds, such as *Aspergillus fumigatus*, a known human pathogen, which may complicate the clinical management of patients suspected to harbor invasive fungal infections [55].

Currently, there are no Jordanian guidelines on the diagnosis, management, and treatment of IFI; rather, scattered efforts are guided by several international societies. Authors from the Arabian Peninsula and Lebanon convened in Dubai in 2014 and released guidelines on the management and treatment of invasive candidiasis and aspergillosis, based on the international literature and guidelines, supported by limited local data [56,57,58]. Stephens and her coworkers published in 2017 an evaluation of the burden and treatment patterns of IFI in hospitalized patients in the Middle East, studying real-world data; however, the study was limited to Saudi Arabia and Lebanon [59].

In conclusion, awareness of the burden of fungal infections by the healthcare stakeholders in Jordan, to include fungal infections in the official Ministry of Health surveillance registry, and to urge hospitals through the Health Care Accreditation Council (HCAC) to include fungal infections and their susceptibility in a reporting system, comparable to bacteria and their drug resistant patterns, is necessary. Worldwide, continued efforts are needed at many levels to have their share in better fungal diagnostic methods to guide early intervention and management, as well as controlling resistance by prohibiting the use of anti-infective agents, including antifungals in the food industry. Those concerned levels include, but are not limited to, ministries of health, international organizations, the pharmaceutical industry, healthcare professionals, and stakeholders in agriculture, veterinarians, and the livestock industry.

## Figures and Tables

**Table 1:** Total burden of fungal infections per the underlying disorder and per year in Jordan.

Infection	Number of Infections Per Underlying Disorder/Year					Total Burden	Rate/100K	Default Assumptions Used and Reference
	None/Other	HIV/AIDS	Respiratory	Cancer/Tx ^a^	ICU			
Invasive/systemic/deep infections						629	7.7	-
Candidemia	-	-	-	221	95	316	5.0	[3]
*Candida* peritonitis	-	-	-	-	47	47	0.75	[33]
Mucormycosis	-	-	-	1	-	1	0.02	-
Cryptococcal meningitis	-	0	-	0	-	-	0	-
*Pneumocystis* pneumonia	-	6	-	-	-	6	0.1	-
IA ^b^	-	-	-	39	45	84	1.34	[17]
CPA ^c^	-	-	175	-	-	175	11	[25]
Allergic fugnal disease						20,648	327	-
ABPA ^e^	-	-	8900	-	-	8900	141	[25]
SAFS ^f^	-	-	11,748	-	-	11,748	186	-
Superficial infections often requiring systemic treatment						97,876	-	-
Oral Candidiasis	-	9	-	-	-	9	0.14	EMRO ^d^ 2012 [15,20,21,23]
Oesophageal candidiasis	-	6	-	56	-	62	1.0	[17,19]
Recurrent *Candida* vaginitis (>4x/year)	97,804	-	-	-	-	97,804	3097 *	[25]
Total burden estimated	97,804	21	20,823	317	187	119,153	1887	-

- No estimate made or possible; ^a^ Treatment; ^b^ Invasive aspergillosis. ^c^ Chronic pulmonary aspergillosis; ^d^ East Mediterranean Regional Office; ^e^ Allergic bronchopulmonary aspergillosis; ^f^ Severe asthma with fungal sensitization. Tx = transplant recipients; * Females only; NA = not. Total Burden: the total absolute calculated cases based on population number and rate for the year.

**Table 2:** The burden of serious fungal infections in selected Arab countries located in Southwest Asia and North Africa.

	Iraq [29]		Saudi Arabia [28]		Egypt [30]		Algeria [31]	
	Burden	Rates	Burden	Rates	Burden	Rates	Burden	Rates
Candidemia	1700	5	2808	10	417	5	2020	5
*Candida* peritonitis	-	-	444	1.6	619	0.75	303	0.75
Oral candidiatis	90	0.26	-	-	2250	2.73	-	-
Esophageal candidiasis	48	0.14	-	1.4	700	0.85	832	2.1
Recurrent *Candida* vaginitis	453,000	2664	466,133	33.20	1,307,766	3168.92	485,188	2402
Invasive aspergillosis	892	2.62	2146	7.6	8832	10.7	2865	7.1
Allergic bronchopulmonary aspergillosis	5421	16	59,466	212	133,834	162.15	31,310	77
Severe asthma with fungal sensitization (SAFS)	4363	13	78,495	280	176,661	214.04	41,329	102
Chronic pulmonary aspergillosis	389	1.15	965	3.4	3015	13.83	897	2.2
Mucormycosis	68	0.2	10	0.034 ^a^	-	-	79	0.2
Pneumocystis pneumonia	1	0	-	-	125	0.15	74	0.18
Cryptococcal meningitis	0	0	-	-	38	0	790	0.09
Fungal keratitis	-	-	-	-	11,550	14		
Tinea capitis	65,200	192	-	-	-	-	4265	10.6

- No estimate made or possible. Burden: the total absolute calculated cases based on population number and rate for the year. ^a^ Calculated from their burden and population count.

**Table 3:** Burden of serious fungal infections of some world countries calculated as per 100,000 population.

	Vietnam [44]	Mexico [45]	Guatemala [46]	Nigeria [47]	Tanzania [48]	Senegal [40]	UK [42]	China [41]	Spain [43]	Australia [50]	Canada ^g^ [34]
Candidemia	5	5	5	6	5	-	8.14	5	10.7	1.87	4.9
Esophageal Cryptococcosis	36	-	29	93.2	203	14	0.07–0.58	3.7	23.9	-	22.37
*Candida* peritonitis	-	3.5	1.4	1.5	0.75	-	0.14 ^f^	1.4	-	-	0.74
IA	15.99	4.47	4.4	0.6	0.05	-	4.59–4.61	11.9	2.86	3-29 ^c^	1.9
CPA	61	15.9	9.6	78	24	19	0.32–5.7	19.5	9.18	-	4.26
ABPA	26	60	36	60.5	44	71	175–372	36.1	126	245	172
SAFS	34	-	48	79.9	57	93	192–654	47.6	198	323	277
rVVC ^d^	3893	5999	2894	3800	3482	2712	-	2929	874	6212	1403
Mucormycosis	0.12	0.1	-	0.2	-	0.2	0.09	0.2	0.2	0.13 ^a^	0.12 ^e^
Cryptococcal meningitis	0.15	3.4	2.2	37.4	14.66	2.6	0.16	0.17	0.03	6.4 ^b^	0.18
*Pneumocystis jirovecii* Pneumonia	0.67	4.5	4.7	48.2	22	8.2	0.330–00.93	1.8	3.4	-	0.19

- No estimate made or possible. ^a^ Non-*Aspergillus* molds. ^b^ Cryptococcosis. ^c^ Including other forms of invasive fungal disease. ^d^ Recurrent vulvovaginal candidiasise. ^e^ Authors’ inference from the literature. ^f^ Calculated for (chronic ambulatory peritoneal dialysis) CAPD as a risk group only. ^g^ Calculated from Canada burden (absolute numbers) and the population count.
